# Editorial: Metaflammation in obesity and diabetes

**DOI:** 10.3389/fendo.2024.1540999

**Published:** 2025-01-16

**Authors:** Suprabhat Mukherjee, Rakesh Kundu, Melita Vidaković

**Affiliations:** ^1^ Integrative Biochemistry & Immunology Laboratory (IBIL), Department of Animal Science, Kazi Nazrul University, Asansol, West Bengal, India; ^2^ Cell Signaling Laboratory, Department of Zoology, Visva-Bharati (A Central University), Santiniketan, West Bengal, India; ^3^ Molecular Biology Department, Institute for Biological Research, Belgrade, Serbia

**Keywords:** metaflammation, obesity, diabetes, toll-like receptor 4, inflammation, immunoendocrinology

Lifestyle diseases are indeed becoming life-threatening. Obesity and diabetes are now considered to be two major inflammatory diseases that have been disguised over the years as only metabolic diseases. Now there is a growing interest in dissecting the role of metaflammation and inflammation as a cause and/or consequence for its direct contribution to the pathogenesis and/or pathophysiology of these life-threatening diseases. Since the last decade, cutting-edge research has identified obesity as one of the key mediators and predisposing factors in the context of inflammatory pathogenesis of diabetes and beyond, including cancer. Intriguingly, obesity has been presented as the promoter of diabetes as well as cancer through the experimental and clinical research conducted throughout the last 10 years (1, 2). Keeping this in mind, the present Research Topic was conceived with the aim of presenting the current updates on topics ranging from the mechanistic insights of inflammatory consequences induced in obesity, signaling defects/dysregulation in cell death pathways and endocrine networks, responses to infectious agents, and more, all relevant to the clinical progression of diabetes. A total of 7 contributions were received from 8 countries, including 4 original research articles and 3 review articles. All 3 reviews and one research article described the mechanistic insights of metaflammation in diabetes and possible therapeutic options while the rest of the articles focused on obesity.

Low-grade persistent inflammation in obesity is linked to a number of serious health setbacks including diabetes. In compliance with the aim of the present Research Topic, the study by Uroić et al. has revealed a significantly close association between the expression of chemokine ligand/receptor and pathological abnormalities of obesity along with obesity-induced diabetes i.e., an abundance of CCR4^+^ T lymphocytes vs vascular inflammation, CXCR4^+^ subsets vs albuminuria, and CXCR3^+^ T lymphocytes vs dyslipidemia and these chemokine axes have been proposed as potential therapeutic targets. In addition, obesity due to overfeeding during the early postnatal period has been demonstrated to signal polycystic ovary syndrome (PCOS) and insulin resistance by evoking proinflammatory responses through activation of NLRP3 followed by an increase in IL-1β expression and deactivation of AMPK as experimentally documented by Veličković et al. In healthy individuals, anti-inflammatory mediators reverse pro-inflammatory episodes. Adiponectin (ADP) synthesized in adipocytes is known to act as an anti-inflammatory adipokine that links lipid metabolism and glucose homeostasis to regulate energy balance and insulin secretion to prevent obesity (3). However, a cross-sectional study by Nielsen et al. has demonstrated a negative regulation between ADP levels and insulin resistance in cystic fibrosis- related diabetes (CFRD).

Diabetes, especially T2DM is a multifactorial disease and is regulated at multiple levels including insulin secretion, inflammatory cytokine levels, structural integrity and function of adipocytes and pancreatic islets. Under homeostatic conditions, pancreatic islets or mainly β cell function are critically regulated by autophagy while autophagy is inhibited by mTORC1 and promoted by LC3 and p62/SQSTM1, REV-ERBα (Lee, 2014). Hyperglycemia/glucotoxicity-driven perturbation in autophagy results in distortion of islet integrity, insulin resistance and other complications that could be addressed by a series of chemotherapeutics (Metformin, Liraglutide, vitamin D3 and B6) and phytochemicals (Kaempferol, Silymarin), especially traditional Chinese medicine (Yunpi Heluo decoction (YPHLD), Xiaokeping (XKP)), as critically reviewed by Zhao et al. in this Research Topic.

Complications in T2DM include functional abnormalities in important organs like the pancreas, heart, kidney, liver, eye, skin etc. In this context, matrix metalloproteinases (MMPs) are known to promote the morbidities and complications of diabetes like nephropathy and neuropathy (4). Therefore, the roles of tissue inhibitors of metalloproteinases (TIMs) are indeed interesting. Here, the attenuating role of TIMP3 overexpression in MacT3 mice model of Type 1 diabetes mellitus (T1DM) has been experimentally documented by Casagrande et al. The mechanism of protection was described to be orchestrated through the reduction of insulitis and serum levels of the pro-inflammatory cytokines such as TNF-α, IL-1β, and IFN-γ. These inflammatory mediators also promote many complex immunological consequences that potentially include NETosis i.e. the generation of Neutrophil extracellular traps (NETs) (5). Zhu et al. have reviewed the mechanism of NETosis in diabetes and they emphasized the positive correlation between the sero-abundance of NET products (neutrophil elastase, myeloperoxidase, PKC) in diabetic individuals and pathological complications like delayed wound healing, retinopathy, and atherosclerosis. Interestingly, a number of synthetic (metformin, ruboxistaurin) and natural small molecules (vitamin D, silybin) have been highlighted as possible remedies to be verified through clinical trials. In addition to these, Aravindhan and Yuvaraj have summarized the research findings on the anti-inflammatory milieu of latent mTB infection and the subsequent reduction of metaflammation along with the risk of insulin resistance.

T2D and obesity are both associated with metabolic syndrome, which is characterized by chronic low-grade inflammation (metaflammation) in tissues responsible for energy homeostasis, including adipose tissue, liver and pancreatic islets. In particular, the metabolic consequences of adipose tissue dysfunction increase mortality in patients with T2D, underscoring the importance of metaflammation in the context of T2D (6). Recent trends in metaflammation highlight its central role in obesity and diabetes, with an emerging focus on weight cycling, gut microbiome, adipose tissue signaling, and immune cell crosstalk as key drivers. Weight cycling disrupts body composition, leading to rapid fat regain and delayed muscle recovery. Persistent metaflammation in adipose and muscle tissue is believed to drive this phenomenon, creating a state of chronic low-grade inflammation that is challenging to resolve. As this systemic inflammation spreads, it exacerbates the metabolic complications linked to weight cycling (7). Addressing weight cycling may require targeting the root causes of metabolic inflammation and improving the immune microenvironment of adipose and muscle tissue as a foundation step toward prevention and recovery. Furthermore, in the context of metabolic inflammation, the mechanisms by which excessive nutrition triggers oxidative stress in adipose tissue and why this oxidative stress provokes an inflammatory response remain unexplored. ROS-initiated pro-inflammatory signaling to YAP/TAZ-mediated transcriptional regulation may be another pathway of interest but the involvement of this pathway in inflammation is still poorly understood, despite its significance in unraveling the pathogenesis of metaflammation.

**Figure 1:**
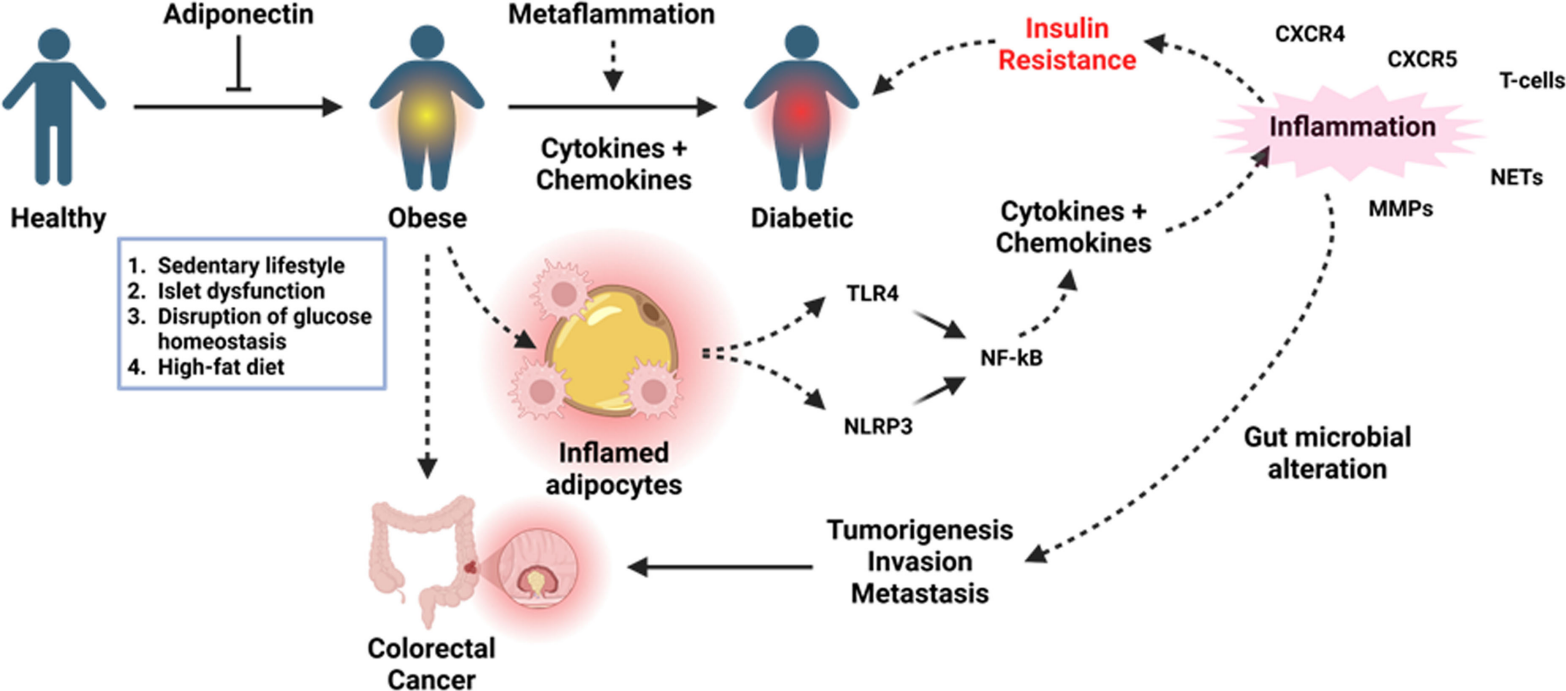
Metaflammation in obesity and its overall impact on the inflammatory and endocrine homeostasis in the context of diabetes and colorectal cancer. Sustained inflammation resulting from high-fat diet-induced free fatty acids can result in high levels of proinflammatory cytokines and chemokines to cause insulin resistance contributing to diabetes, whilst proinflammatory cytokines drive tumorigenesis through the inflammation-dysplasia-neoplasia sequence. Created with https://BioRender.com.

The prevalence of obesity is indeed increasing due to changes in lifestyle, dietary habits and environmental changes, g and new fatal outcomes are being reported from both developed and developing countries. In particular, obesity-induced cancer is also threatening the world with the increasing prevalence of its different subtypes (8), especially colorectal cancer. Interestingly, obesity-induced inflammation and further complications described in this editorial are mainly linked to Toll-like receptor 4 (TLR4) as summarized in **Figure 1**. Although this type-I transmembrane glycoprotein receptor is indispensable in conferring immunity to pathogens and regulating immunity to cancer (9, 10) dysregulated activation of this receptor disrupts the inflammatory homeostasis and this is a critical mediator in inducing pathology in most of the inflammatory diseases and syndromes (8–10) including but not limited to diabetes and colorectal cancer. Therefore, inflammatory mediators like TLR4 could be a potential target to mitigate these health setbacks.
